# Tripeptide binding in a proton‐dependent oligopeptide transporter

**DOI:** 10.1002/1873-3468.13246

**Published:** 2018-09-21

**Authors:** Maria Martinez Molledo, Esben M. Quistgaard, Christian Löw

**Affiliations:** ^1^ Centre for Structural Systems Biology (CSSB) DESY and European Molecular Biology Laboratory Hamburg Hamburg Germany; ^2^ Department of Medical Biochemistry and Biophysics Karolinska Institutet Stockholm Sweden; ^3^ Present address: Department of Molecular Biology and Genetics – DANDRITE Aarhus University Gustav Wieds Vej 10 DK‐8000 Aarhus C Denmark

**Keywords:** major facilitator superfamily (MFS), membrane protein crystallography, peptide binding, peptide transporters, POTs

## Abstract

Proton‐dependent oligopeptide transporters (POTs) are important for the uptake of di‐/tripeptides in many organisms and for drug transport in humans. The binding mode of dipeptides has been well described. However, it is still debated how tripeptides are recognized. Here, we show that tripeptides of the sequence Phe‐Ala‐Xxx bind with similar affinities as dipeptides to the POT transporter from *Streptococcus thermophilu*s (PepT_S__t_). We furthermore determined a 2.3‐Å structure of PepT_S__t_ in complex with Phe‐Ala‐Gln. The phenylalanine and alanine residues of the peptide adopt the same positions as previously observed for the Phe‐Ala dipeptide, while the glutamine side chain extends into a hitherto uncharacterized pocket. This pocket is adaptable in size and can likely accommodate a wide variety of peptide side chains.

## Abbreviations

**DDM,** n‐dodecyl‐β‐D‐maltoside

**DSF,** differential scanning fluorimetry

**LCP,** lipid cubic phase

**MFS,** major facilitator superfamily

**MST,** microscale thermophoresis

**PepT**_**St**_ **,** POT from the bacterium *Streptococcus thermophilus*

**POT,** proton‐dependent oligopeptide transporter

Proton‐dependent oligopeptide transporters (known as POTs, PTRs, or PepTs) are present in all organisms from bacteria to humans 1 where they play key roles in the uptake of dietary di‐ and tripeptides 2, 3. Furthermore, human POTs (PepT1 and PepT2) also act as vehicles for the uptake of peptidomimetic drugs and amino acid‐coupled prodrugs 4, 5. POTs belong to the major facilitator superfamily (MFS), and thus contain two canonical MFS domains, between which the binding site is located 6, 7. During the transport cycle, these domains move relative to each other to allow alternate access from the cytoplasmic and extracellular sides 6. Several X‐ray structures of bacterial POTs have been reported, either in the apo‐form 8, 9, 10, 11, 12, 13, 14, 15, 16, peptide bound state 17, 18, 19, 20, or in complex with the phosphonodipeptide alafosfalin 21, 22 (Table S1). However, only three structures have hitherto been reported in which a tripeptide is bound. These include two structures of PepT_So2_ from *Shewanella oneidensis* in complex with Ala‐Ala‐Ala (PDB ID 4TPJ) and Ala‐Tyr(Br)‐Ala (PDB ID 4TPG) (‘Br’ denotes that the residue is brominated) 19, and a single structure of PepT_St_ from *Streptococcus thermophilus* in complex with Ala‐Ala‐Ala (PDB ID 4D2D) 17. In the PepT_So2_ structures, both peptides were found to extend horizontally across the binding cavity, as also observed for all dipeptides 19. However, as the resolution was rather low (3.2 Å for Ala‐Ala‐Ala and 3.9 Å for Ala‐Tyr(Br)‐Ala), the peptide backbone geometry and binding mode could not be described in detail (Fig. S1A–C). The structure of PepT_St_ in complex with Ala‐Ala‐Ala was determined at a moderately high resolution of 2.5 Å, and suggested an alternative vertical orientation of the peptide (Fig. S1D–F). However, in a recent effort to characterize binding of different dipeptides to PepT_St_, we obtained data suggesting that the observed vertically bound molecule in PepT_St_ might instead have been a misidentified HEPES buffer molecule 18. There is, therefore, an outstanding need for more structural insights into how tripeptides are recognized by POTs. Transport competition assays and binding studies have suggested that PepT_St_ prefers dipeptides over tripeptides, although only a tiny subset of the 8000 possible tripeptide sequences were tested, namely Ala‐Ala‐Ala, Ala‐Pro‐Ala, Leu‐Leu‐Ala, Ala‐Phe‐Ala, and Ala‐Leu‐Ala 8, 18. In our previous study of PepT_St_, we noted that dipeptides can bind in at least two different overlapping positions, one represented by Ala‐Leu, Ala‐Gln, and Asp‐Glu (binding mode 1), and another represented by Phe‐Ala (binding mode 2) (Fig. S1G–I) 18. Furthermore, these binding modes correlate with different positions of Tyr‐68 8, 18, 23. This residue thus partially blocks the remainder of the binding cavity in binding mode 1, but not in binding mode 2 where it seems there would be considerably more space for accommodating a third residue added at the C terminus (Fig. S2A–F). Therefore, we hypothesized that tripeptides designed as being C‐terminally extended versions of dipeptides binding in mode 2, that is, tripeptides with a Phe‐Ala‐Xxx sequence would be able to bind well to PepT_St_.

## Materials and methods

### Protein purification and peptide stocks

For WT PepT_St_, the same construct was used as reported previously 24. Mutations were introduced by blunt end PCR and confirmed by sequencing. Expression and purification of PepT_St_ WT and PepT_St_ mutants were carried out as previously described 15, 18, 24. The peptides used in this study were chemically synthesized and acquired from GL Biochem (Shanghai). The lyophilisates were dissolved in water or DMSO at a concentration of 100 mm.

### Stability measurements

PepT_St_ transition midpoint (Tm) in the presence or absence of tripeptides was measured using the nanoDSF Prometheus NT.48 device (NanoTemper technologies). Here, the change in the intrinsic fluorescence of the protein was recorded at 330 and 350 nm over a temperature ramp of 20–90 °C. The Tm value was calculated from the first derivative of the unfolding curves. Each run was performed as described in 18. The peptides were tested at concentrations of 0.625, 1.25, 2.5, and 5 mm. For stability measurements, microscale thermophoresis (MST), and crystallization, the pH was a key parameter to control, as explained previously 18. The pH of the peptide stock solutions was in the range of 2.0–2.5. Measurements were, therefore, performed in 100 mm HEPES/NaOH at pH 7.5 to maintain the pH constant regardless of the peptide concentration. The buffer furthermore contained 150 mm NaCl and 0.4% n‐nonyl‐β‐D‐maltoside (NM), except for the comparison of PepT_St_ WT with the various mutants, where NM was replaced by 0.03% n‐dodecyl‐β‐D‐maltoside (DDM), which markedly enhances the thermal stability of the protein 15.

### Binding measurements

Peptide binding to detergent‐solubilized PepT_St_ (WT or single mutants) was measured with the Monolith NT. LabelFree MST device (NanoTemper technologies) 18, 25, using a protein concentration of 125 nm and a highest peptide concentration of 50 mm. The measurements were performed in 400 mm HEPES/NaOH pH 7.5, 150 mm NaCl, and 0.03% DDM at 22 °C, 15–20% LED power and 20% MST power. Binding curves were plotted and analyzed using GraphPad Prism (GraphPad Software, San Diego, CA), assuming a 1 : 1 binding stoichiometry.

### Crystallization

Crystallization was carried out in lipid cubic phase (LCP) 26 in essentially the same way as described for other PepT_St_[peptide] complexes 17, 18. The crystallant contained 100–300 mm HEPES buffer, 250 mm NH_4_H_2_PO_4_, 15–25% PEG 400, and 30 mm of the cocrystallized peptide. The pH was kept in the range of 5.5–6.5 in order to both favor peptide binding and promote protein crystallization 18. Crystals generally appeared within 24 h, grew further during the following 3 days, and were harvested and flash frozen in liquid nitrogen after 7–10 days.

### Data collection and structure determination

Crystal screening and data collection were performed at the P14 beamline at the PETRA III storage ring (c/o DESY, Hamburg, Germany) (see Table 1). The data were processed using XDS 27, and the initial models were obtained by molecular replacement, performed with the Phaser program in the PHENIX package 28. Several cycles of refinement with phenix.refine 29 and manual model building in Coot 30 were used to obtain the structures. Simple peptide omit maps were generated by repeating the last round of refinement, omitting the bound peptides in the inputted PDB file. Simulated annealing composite omit maps were generated in Phenix with the omitted fraction set at the default value of 0.05. PyMol (Schrödinger LLC; http://www.pymol.de) was used for generating structure figures, and LigPlot^+^ 31 for generating the PepT_St_[Phe‐Ala‐Gln] interaction diagram.

**Table 1:** Crystallographic data processing and refinement statistics for PepT_St_ in complex with Phe‐Ala‐Xxx tripeptides. Numbers in parentheses refer to the highest resolution shell

	PepT_St_[Phe‐Ala‐Gln]	PepT_St_[Phe‐Ala‐Ala]	PepT_St_[Phe‐Ala‐Thr]
Data collection			
Beamline	PETRA III P14	PETRA III P14	PETRA III P14
Wavelength (Å)	0.9143	0.9762	0.9762
Space group	C222_1_	C222_1_	C222_1_
Cell dimensions			
*a*,* b*,* c* (Å)	101.55, 108.22, 111.61	102.27, 108.99, 112.39	101.02, 108.05, 110.50
α, β, γ (°)	90, 90, 90	90, 90, 90	90, 90, 90
Resolution (Å)	48.69–2.26 (2.34–2.26)	49.03–2.00 (2.07–2.00)	48.53–2.10 (2.18–2.10)
*R*_merge_	0.083 (0.811)	0.073 (0.786)	0.096 (1.708)
*I/*σ*I*	19.44 (3.19)	16.32 (2.23)	12.29 (1.03)
CC1/2	0.999 (0.871)	0.999 (0.776)	1 (0.468)
Completeness (%)	99.73 (99.79)	99.74 (99.83)	99.59 (98.49)
Total no. reflections	299 776 (29 604)	294 024 (29 583)	241 819 (23 429)
Multiplicity	10.3 (10.4)	6.9 (7.0)	6.9 (6.8)
Wilson *B*‐factor (Å^2^)	40.59	34.54	46.82
Refinement			
*R*_work_/*R*_free_	0.193/0.219	0.189/0.215	0.208/0.237
No. atoms			
Protein	3529	3636	3527
Bound tripeptide	26	22	24
HEPES/PEG/ions	41	39	36
Lipids	308	264	264
Water	101	171	85
*B*‐factors			
Protein	48.66	38.81	58.30
Bound tripeptide	51.94	49.07	84.81
HEPES/PEG/ions	96.49	79.05	95.88
Lipids	82.60	65.52	82.72
Water	53.04	46.08	56.94
R.m.s. deviations			
Bond lengths (Å)	0.004	0.003	0.008
Angles (°)	0.75	0.62	0.82
Ramachandran			
Favored (%)	98.89	98.92	98.66
Outliers (%)	0.00	0.00	0.00
Clash score	5.22	2.45	5.30
PDB accession	6GHJ	–	–

The atomic coordinates and structure factors for PepT_St_ in complex with Phe‐Ala‐Gln have been deposited in the Protein Data Bank with accession number PDB: 6GHJ.

## Results and discussion

### Characterization of tripeptide binding to PepT_St_

**Figure 1:**
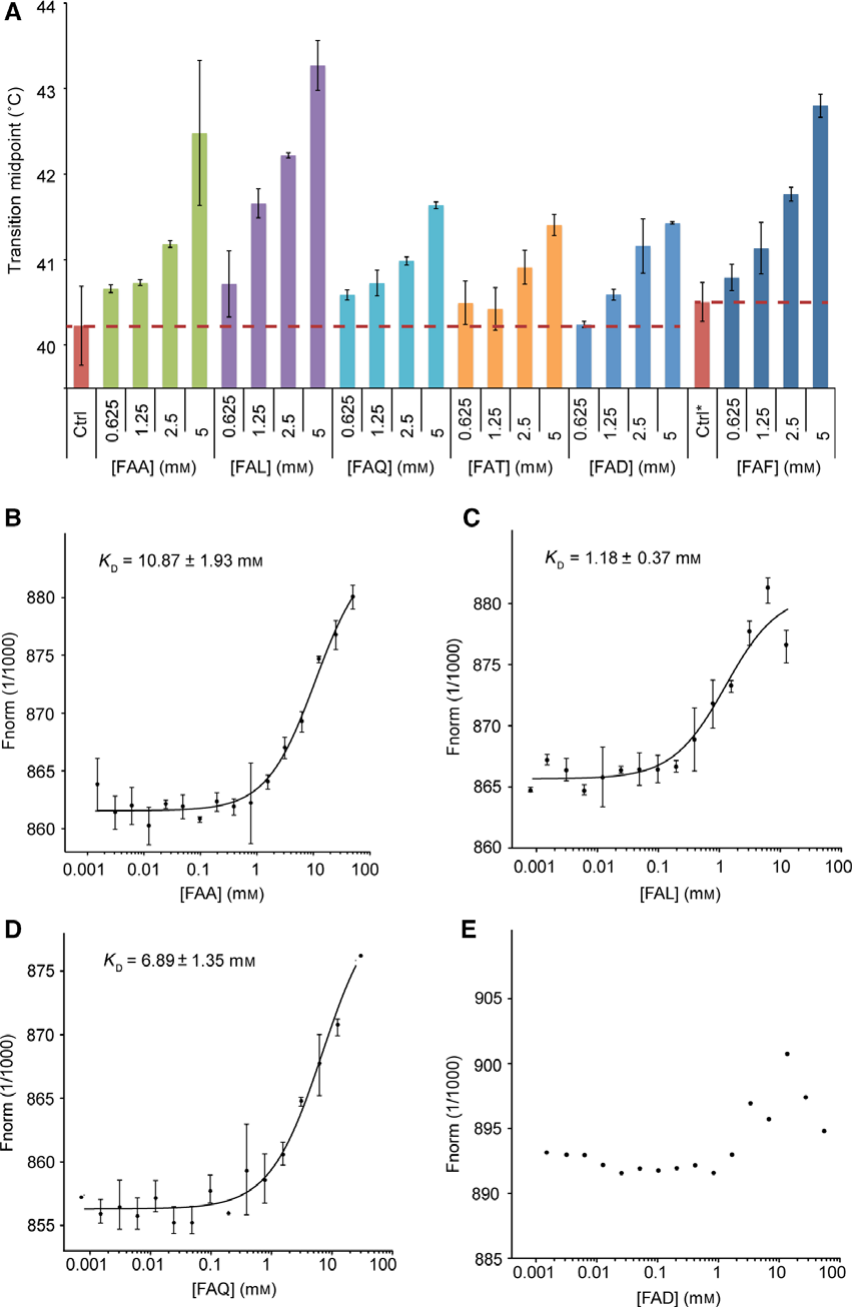
Binding of Phe‐Ala‐Xxx tripeptides as measured by nanoDSF and MST. (A) Thermostability data for PepT_S__t_ measured by nanoDSF. Each Phe‐Ala‐Xxx tripeptide was measured at four different concentrations: 0.625, 1.25, 2.5, and 5 mm, as indicated on the *x*‐axis. Results for control samples, which did not contain any tripeptide, are shown as red bars (Ctrl is without DMSO, and Ctrl* is with 5% DMSO). For these samples, the transition midpoint (Tm) is further indicated by red dashed lines. Results for the tripeptides are shown as differently colored bars: Phe‐Ala‐Ala is light green, Phe‐Ala‐Leu is purple, Phe‐Ala‐Gln is light blue, Phe‐Ala‐Thr is orange, Phe‐Ala‐Asp is blue, and Phe‐Ala‐Phe is dark blue. Note that Phe‐Ala‐Phe should be compared to Ctrl* rather than Ctrl, since it was solubilized in DMSO. The average Tm value for each condition was calculated from three independent measurements. The error bars correspond to the standard deviation from these independent measurements. (B) MST binding curve for Phe‐Ala‐Ala. Error bars represent the standard deviation of two independent measurements. The estimated dissociation constant (*K*_D_) is indicated. (C) MST binding curve for Phe‐Ala‐Leu (shown as for panel B). (D) MST binding curve for Phe‐Ala‐Gln (shown as for panel B). (E) MST binding curve for Phe‐Ala‐Asp. Here, the binding was too weak to allow a *K*_D_ value to be determined.

We used differential scanning fluorimetry (DSF) 32 to study the binding of Phe‐Ala‐Ala, Phe‐Ala‐Leu, Phe‐Ala‐Gln, Phe‐Ala‐Thr, Phe‐Ala‐Asp, and Phe‐Ala‐Phe to detergent‐solubilized PepT_St_ (Fig. 1A). These peptides were all found to stabilize the protein against thermal unfolding and aggregation, which is indicative of binding. The strongest effect was observed for peptides with a bulky apolar residue in the third position, that is, Phe‐Ala‐Leu and Phe‐Ala‐Phe. These two peptides were also more stabilizing than any of the previously tested tripeptides, although markedly less compared to the best performing dipeptides 18. Next, we used MST 25 to further characterize the binding of Phe‐Ala‐Xxx peptides to PepT_St_ (Fig. 1B–E). Here, we obtained the following dissociation constants: *K*_D_ (Phe‐Ala‐Ala) = 10.87 ± 1.9 mm (Fig. 1B), *K*_D_ (Phe‐Ala‐Leu) = 1.18 ± 0.3 mm (Fig. 1C), *K*_D_ (Phe‐Ala‐Gln) = 6.89 ± 1.3 mm (Fig. 1D), and *K*_D_ (Phe‐Ala‐Thr) = 28.09 ± 12.3 mm (data not shown). In the case of Phe‐Ala‐Asp, binding was too weak for a *K*_D_ value to be determined (Fig. 1E). The Phe‐Ala‐Phe tripeptide was only soluble in DMSO (100 mm stock), which did not allow us to determine a full binding isotherm using MST. As the Phe‐Ala dipeptide displays a *K*_D_ value of 10.95 ± 2.2 mm 18, we can infer that: (a) Extending this peptide with an extra alanine residue has no effect on the binding affinity, indicating that it can be accommodated in the binding site, but without strengthening the interaction. (b) Adding instead a threonine or aspartate residue moderately or strongly reduces affinity, respectively, suggesting that especially the latter is clashing within the binding site. (c) Extending the peptide with a glutamine residue in the third position improves the affinity slightly, while adding a leucine residue improves it markedly, indicating that, in particular, the latter contributes significantly to the interaction of the tripeptide with the binding site. We conclude that both the DSF and MST results indicate that bulky apolar residues are preferred over smaller polar/charged ones in the third position of the Phe‐Ala‐Xxx‐ peptides. It may also be noted that the affinity of Phe‐Ala‐Leu is higher than for most of the previously tested dipeptides, and only 2.1‐fold lower than for the best binding one, Ala‐Leu, which displayed an affinity of 0.56 ± 0.08 mm 18. Thus, while it is possible that a systematic analysis of all di‐ and tripeptides would bear out the notion that PepT_St_ on average prefers dipeptides, it is evident that some tripeptides also bind quite well.

### Structures of PepT_St_ in complex with Phe‐Ala‐ tripeptides

**Figure 2:**
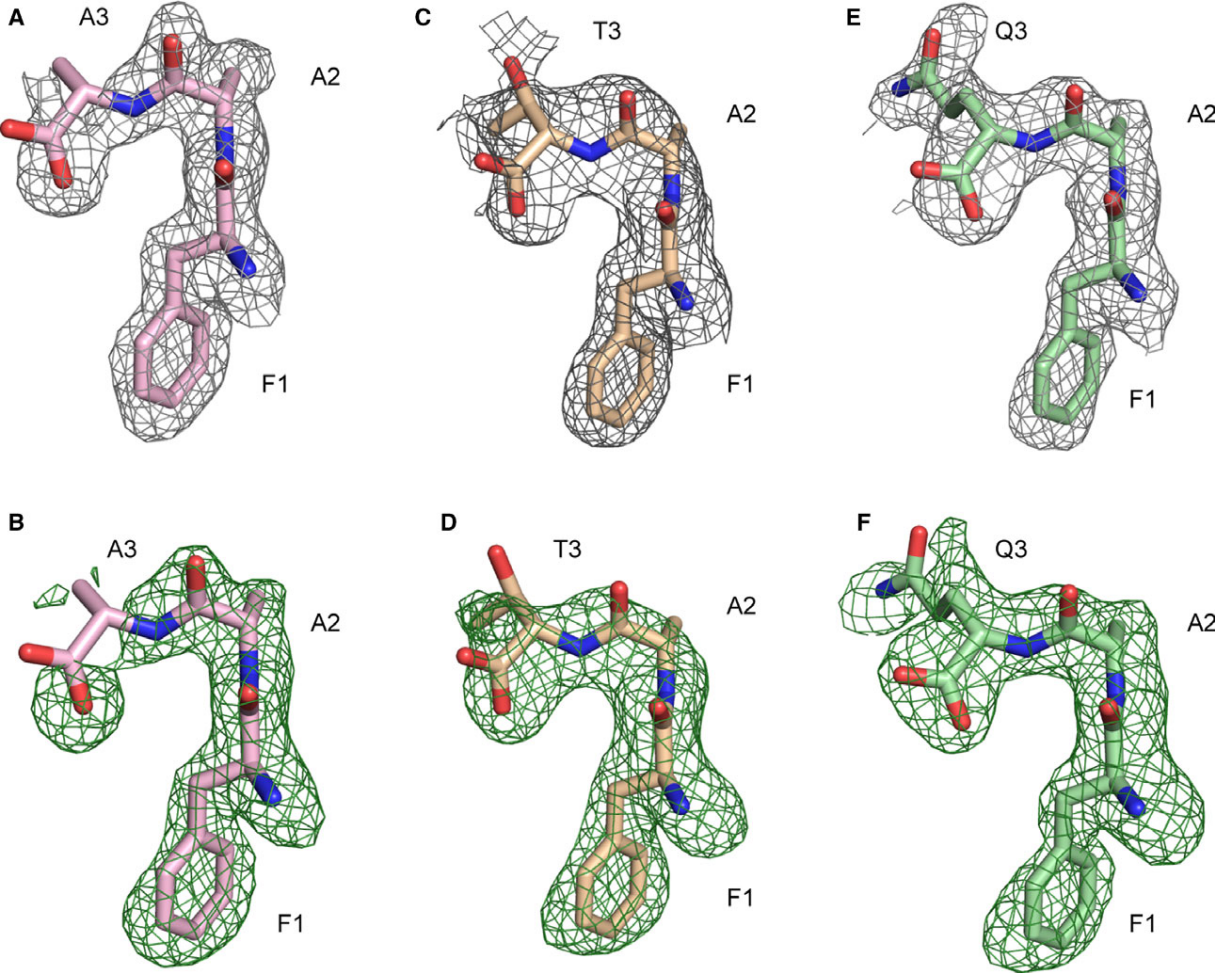
Electron density maps for the bound Phe‐Ala‐Xxx tripeptides. (A) PepT_S__t_ in complex with Phe‐Ala‐Ala (PepT_S__t_[Phe‐Ala‐Ala]). The peptide is shown with the 1‐σ 2Fo‐Fc simulated annealing composite omit map. (B) PepT_S__t_[Phe‐Ala‐Ala] with the 3‐σ Fo‐Fc map generated with the peptide omitted. (C) PepT_S__t_[Phe‐Ala‐Thr] with the 1‐σ 2Fo‐Fc simulated annealing composite omit map. (D) PepT_S__t_[Phe‐Ala‐Thr] with the 3‐σ Fo‐Fc peptide omit map. (E) PepT_S__t_[Phe‐Ala‐Gln] with the 1‐σ 2Fo‐Fc simulated annealing composite omit map. (F) PepT_S__t_[Phe‐Ala‐Gln] with the 3‐σ Fo‐Fc peptide omit map.

To obtain crystals with bound tripeptides, we used the previously published LCP crystallization conditions for PepT_St_ peptide complexes 17, 18. The tripeptide Phe‐Ala‐Leu had a high tendency to form crystals itself under the given conditions, and was, therefore, not used further. As mentioned above, Phe‐Ala‐Phe was poorly soluble in aqueous solutions, and was, therefore, dissolved in DMSO. However, the presence of 5% DMSO was found to have a negative impact on LCP crystallization. Furthermore, attempts to obtain cocrystals by dry coating the plates with this peptide were likewise unproductive. Crystallization succeeded for the following tripeptides: Phe‐Ala‐Ala, Phe‐Ala‐Thr, and Phe‐Ala‐Gln, and high‐resolution diffraction data could be collected for all complexes (maximum resolution ranging from 2.1 to 2.3 Å, see Table 1). The crystal form was the same as that observed for the previously determined structures grown under similar conditions 17, 18. The protein can display either an inward open or an inward facing partially occluded conformation in this crystal form 17, 18. However, in all cases reported here, the conformation was found to be fully inward open. For all three refined structures, clear electron density could be observed for the bound tripeptides in both simple peptide omit maps and in simulated annealing composite omit maps (Fig. 2). It is evident that the peptides all extend across the binding cavity formed in the space between the two MFS domains of the protein, and they thus conform to a horizontal rather than a vertical binding mode (Fig. S3A). It is furthermore evident that the first two residues of all three tripeptides bind in the same way as the two residues of the dipeptide Phe‐Ala (Fig. S3B). However, the C‐terminal residue could not be confidently modeled for Phe‐Ala‐Ala and Phe‐Ala‐Thr. In the former case, the electron density for it was simply too weak (Fig. 2A,B), implying that a C‐terminal alanine residue interacts poorly with the protein, as also indicated by the binding experiments. In the latter case, the electron density map for the C‐terminal residue was stronger, but unfortunately also ambiguous: It was thus possible to flip the positions of the threonine side chain and the C‐terminal carboxylate moiety for each other with no significant adverse effect on refinement and map quality (Fig. 2C,D). For Phe‐Ala‐Gln, it could in contrast be clearly established in which respective directions the side chain and C‐terminal carboxylate moiety are pointing in the binding cavity (Fig. 2E,F). For the remainder of the discussion we will, therefore, focus specifically on this peptide.

### Binding of the backbone of the Phe‐Ala‐Gln tripeptide

**Figure 3:**
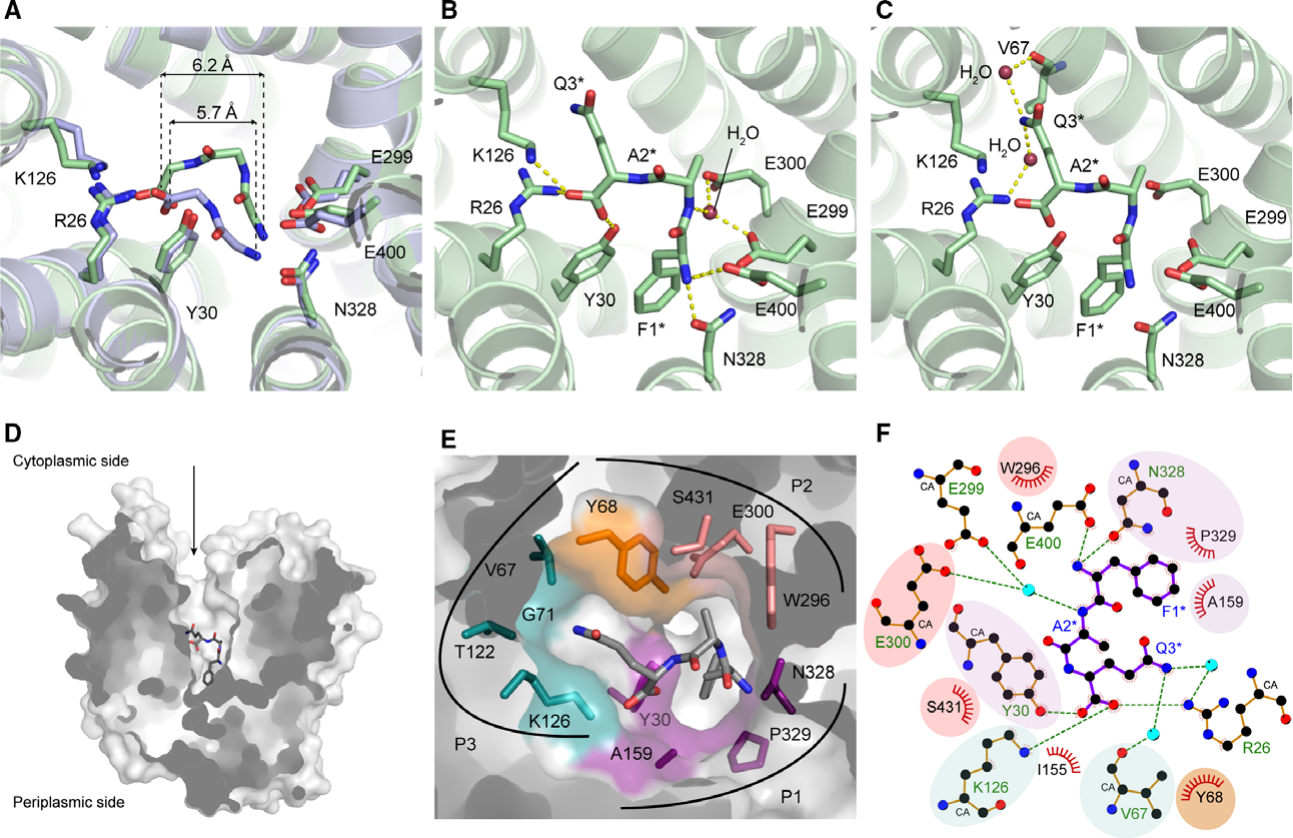
Structural basis for binding of Phe‐Ala‐Gln to PepT_S__t_. (A) Peptide backbone configurations of bound Phe‐Ala‐Gln and Ala‐Leu. PepT_S__t_[Phe‐Ala‐Gln] is light green and PepT_S__t_[Ala‐Leu] is violet. The side chains of selected binding site residues and the backbones of the peptides are shown in sticks and labeled. Distances between the N terminus (nitrogen atom) and C terminus (carboxylate carbon atom) of each peptide are indicated. (B) Interactions with the peptide backbone and termini in PepT_S__t_[Phe‐Ala‐Gln]. Labels followed by an asterisk refer to residues in the peptide. (C) Interactions with the peptide glutamine side chain in PepT_S__t_[Phe‐Ala‐Gln]. (D) PepT_S__t_[Phe‐Ala‐Gln] in surface representation. The arrow indicates the accessibility to the binding cavity from the cytoplasmic side of the membrane (the protein is in the inward open conformation). (E) Binding pockets in PepT_S__t_. The protein is shown in a semitransparent surface representation, but with the residues forming the pockets also shown as sticks. Pocket 1 (P1) is colored purple, pocket 2 (P2) is salmon, pocket 3 (P3) is dark cyan, and Tyr‐68, which is part of both P2 and P3, is orange. The Phe‐Ala‐Gln peptide is shown as gray sticks. (F) LigPlot^+^ diagram for the binding of Phe‐Ala‐Gln to PepT_S__t_. The pockets are indicated by different background colors (using the same color scheme as in panel E). Hydrogen bonds and ionic interactions are indicated by green dashes, and the residues involved are labeled in green. Residues forming hydrophobic contacts to the peptide are labeled in black.

The N‐ and C termini of Phe‐Ala‐Gln interact with the same residues in PepT_St_ as the termini of the dipeptides (Fig. 3A, Fig. S3). Specifically, the N terminus interacts with a subsite consisting of Glu‐299, Asn‐328, and Glu‐400 in the same manner as observed for Phe‐Ala (dipeptide binding mode 2) (Fig. S3B), while the C terminus interacts with a subsite consisting of Arg‐26, Tyr‐30, and Lys‐126 in a similar way as observed for Ala‐Leu (dipeptide binding mode 1) (Fig. S3C). Phe‐Ala‐Gln is able to fit between these subsites in spite of its increased size as compared to a dipeptide because the backbone torsion angles of the central alanine residue fall squarely in the helical region of the Ramachandran plot (φ = −56° and φ = −48°), which enables a highly curved backbone configuration that minimizes the distance between the N‐ and C termini. Indeed, the distance between the N‐terminal nitrogen atom and C‐terminal carboxylate carbon atom is only marginally longer for Phe‐Ala‐Gln than for Ala‐Leu (6.2 Å versus 5.7 Å) (Fig. 3A). Aside from the interactions formed with the termini, the only other backbone interaction is a putative water‐mediated hydrogen bond between the nitrogen atom of the peptide alanine residue and the side chains of Glu‐299 and Glu‐300 (Fig. 3B). However, the electron density for the implicated water molecule is rather weak, suggesting that it is only partially occupied, and therefore probably not of great importance for the interaction with the peptide. To validate the binding site, we used MST to test binding of Phe‐Ala‐Gln to the following five PepT_St_ mutants: R26A, Y30A, E299A, E300A, and E400A. For PepT_St_ R26A, the binding affinity was found to be several fold lower than for the WT protein, while for the other mutants, the binding was found to be so weak that it was not possible to determine a dissociation constant (Fig. S4). The mutagenesis data thus support the observations from the structural analysis. However, it may be noted that the importance of Glu‐300 may relate more to a role in charge balance and/or proton coupling than to its putative water‐mediated interaction with the peptide backbone. A role in proton coupling has thus been found for the equivalent Glu‐310 residue in GkPOT (a POT transporter from the bacterium *Geobacillus kaustophilus*) 22, 33.

### Binding of the side chains of the Phe‐Ala‐Gln tripeptide

Concerning the Phe‐Ala‐Gln side chains, the first two fit into two mostly hydrophobic and aromatic pockets, denoted pocket 1 (P1) and pocket 2 (P2), respectively, in the same way as previously described for the Phe‐Ala dipeptide 18, while the third residue fits into a previously undescribed pocket 3 (P3) (Fig. 3C–F). This pocket neighbors P2, but is partially separated from it by the flexible residue Tyr‐68 (Fig. 3E). We have previously discussed a role for this residue in tuning the size of P2 18, but it is now clear that movements of Tyr‐68 at the same time also affect the dimensions of P3 (Fig. S2). The present work thus further supports the notion that this highly conserved residue (conserved as Tyr‐64 in human PepT1 and Tyr‐94 in human PepT2) plays an important gatekeeper role in the binding site of POTs 23. The side chain of the peptide glutamine residue forms van der Waals interactions with Tyr‐68 and Lys‐126, as well as hydrogen bonds with two water molecules, one of which also interacts with the backbone carbonyl of Val‐67, and the other of which interacts with Arg‐26 (Fig. 3C). However, it should be pointed out that the B‐factors are higher for the glutamine side chain (64), as compared to the other side chains of the peptide (54 for the alanine and 43 for the phenylalanine), as well as the side chains of the interacting binding site residues Tyr‐68 (51) and Lys‐126 (46). This indicates that the glutamine side chain shows some degree of flexibility, and is therefore probably not tightly bound, as also suggested by the binding studies. Other residues delineating P3 include Val‐67, Gly‐71, and Thr‐122 (Fig. 3E,F). As the polar and charged moieties of Thr‐122 and Lys‐126 point away from the pocket, P3 is mostly hydrophobic. Thus, although it can form water‐mediated interactions with polar residues, such as the glutamine side chain of the FAQ peptide, it seems particularly well suited for interacting with apolar residues. An additional property of P3 is that it is quite spacious when Tyr‐68 is in the position observed in this structure, suggesting that it could potentially accommodate quite large side chains. The characteristics of P3 are thus in line with the findings from the binding studies that PepT_St_ can accommodate a wide variety of chemically diverse residues in the third position of Phe‐Ala‐Xxx peptides, but appears to have a preference for bulky apolar ones, such as leucine.

## Conclusion and outlook

We have shown that PepT_St_ can bind Phe‐Ala‐Xxx tripeptides with affinities similar to what has been previously observed for a range of dipeptides, and that Phe‐Ala‐Gln binds to inward open PepT_St_ in a horizontal orientation. This was also observed for the PepT_So2_ tripeptide complexes, however, in those cases, the peptides were much more extended than observed here 19. The structure may facilitate drug design for human PepT1 and PepT2. However, in revealing that the sizes of the side chain‐binding pockets P2 and P3 are tunable via movement of Tyr‐68, it also adds to the emerging picture that the binding sites of POTs have a high degree of plasticity 17, 18, 19, 34, which may make structure‐based drug design a quite challenging task.

## Author contributions

MMM and EMQ contributed equally. All authors have given approval to the final version of the manuscript.

## Data Accessibility

## Supporting information


**Table S1.** Reported bacterial POT structures.
**Fig. S1.** Recapitulation of previous insights into peptide binding in PepT_So2_ and PepT_St_.
**Fig. S2.** Role of Tyr‐68 in di‐ and tripeptide binding in PepT_St_.
**Fig. S3.** Binding mode of Phe‐Ala‐Gln compared to the binding modes of other peptides binding to PepT_St_.
**Fig. S4.** Stability and peptide binding of PepT_St_ mutants.
