# Supplementary material for: Tripeptide binding in a proton‐dependent oligopeptide transporter
Source: FEBS Lett. 2018 Sep 21;592(19):3239–47. doi: 10.1002/1873-3468.13246 (PMC6221056; doi:10.1002/1873-3468.13246)
Supplement: Supplementary file 1 — Table S1. Reported bacterial POT structures. Fig. S1. Recapitulation of previous insights into peptide binding in PepTSo2 and PepTSt. Fig. S2. Role of Tyr‐68 in di‐ and tripeptide binding in PepTSt. Fig. S3. Binding mode of Phe‐Ala‐Gln compared to the binding modes of other peptides binding to PepTSt. Fig. S4. Stability and peptide binding of PepTSt mutants. [file FEB2-592-3239-s001.pdf]

Transporter	Organism	PDB ID	Molecules in the binding site	Resolution (Å)
PepT$_{St}$	*Streptococcus thermophilus*	4APS	-	3.3
		4D2C	Ala-Phe	2.5
		4D2D	Ala-Ala-Ala	2.5
		4D2B	-	2.4
		4XNJ	-	2.3 (cryo)
		4XNI	-	2.8 (RT)
		5MMT	-	3.4
		5D58	Ala-Phe	2.4
		5D59	Ala-Phe	2.4
		5D6K	-	2.4
		5OXL	Ala-Leu	2.7
		5OXK	Ala-Gln	2.4
		5OXM	Asp-Glu	2.3
		5OXN	Phe-Ala	2.2
		6EIA	Phosphate and HEPES	2.0
		5OXQ	Phosphate and HEPES	2.2
		5OXP	Phosphate and PEG	2.4
		5OXO	-	2.0
PepT$_{So2}$	*Shewanella oneidensis*	4LEP<br>4TPG<br>4TPH<br>4TPJ	Alafosfalin<br>Ala-Tyr(Br)-Ala<br>Ala-Tyr(Br)<br>Ala-Ala-Ala	3.2<br>3.9<br>3.2<br>3.2
PepT$_{So}$	*Shewanella oneidensis*	4UVM<br>2XUT	-	3.0<br>3.6
YbgH	*Escherichia coli*	4Q65	-	3.4
YePEPT	*Yersinia enterocolitica*	4W6V		3.0
GkPOT	*Geobacillus kaustophilus*	4IKV<br>4IKW<br>4IKX<br>4IKY<br>4IKZ	-<br>Sulfate<br>-<br>Sulfate<br>Alafosfalin	1.9<br>2.0<br>2.3<br>2.1<br>2.4
PepT$_{Xc}$	*Xanthomonas campestris*	6EI3	-	2.1

Table S1: Reported bacterial POT structures. For each structure, the following information is given: Name of the transporter, organism of origin, PDB ID, the molecules bound in the binding cavity, excluding solvent molecules, and the resolution of the structure.

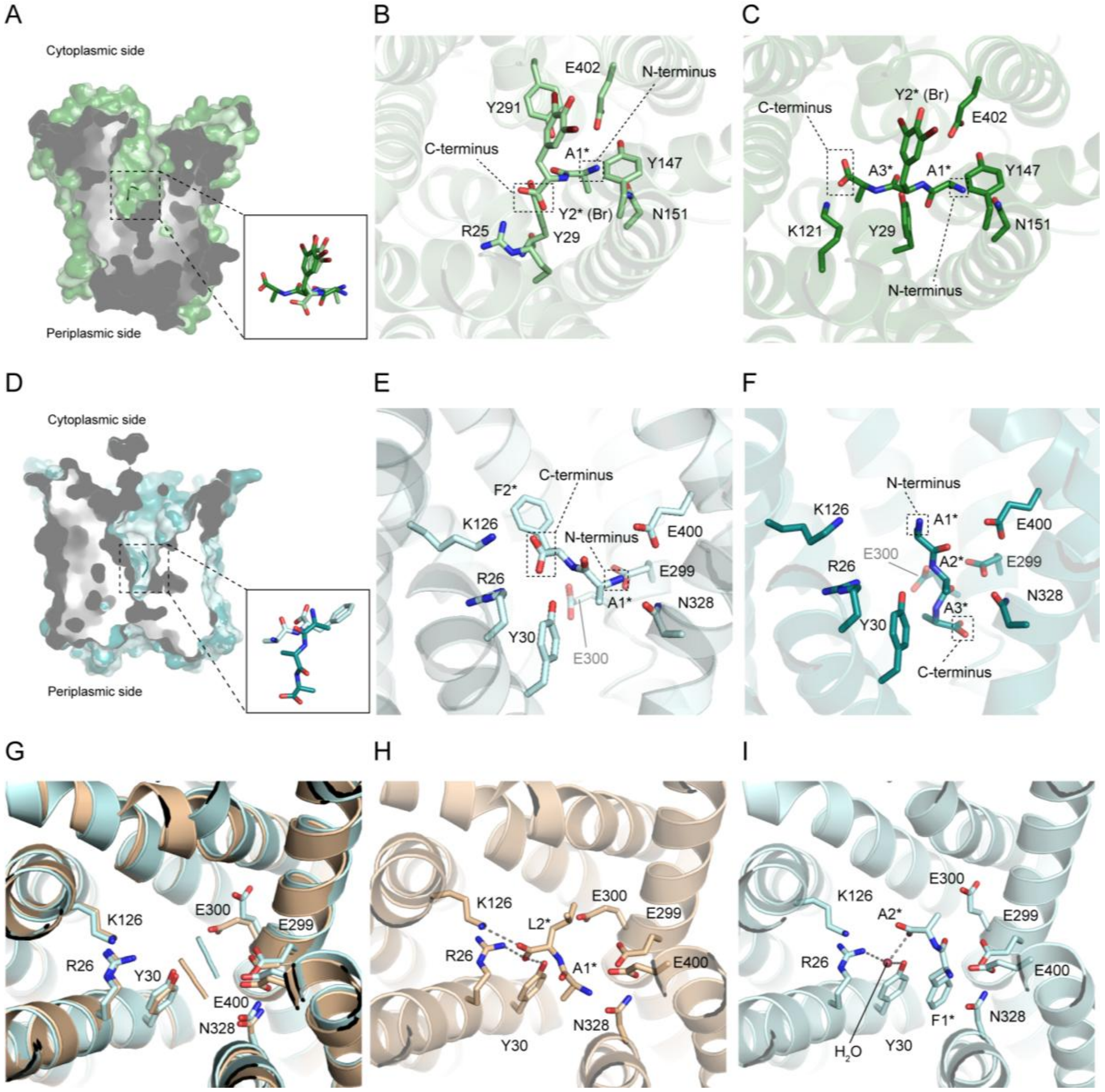

Figure S1: Recapitulation of previous insights into peptide binding in PepT$_{So2}$ and PepT$_{St}$. (A) Surface representation of an overlay of PepT$_{So2}$ in complex with Ala-Tyr(Br) (PDB ID 4TPH) (light green) and Ala-Tyr(Br)-Ala (PDB ID 4TPG) (dark green). The zoomed view in the inset shows the overlap in position of the dipeptide Ala-Tyr(Br) and the tripeptide Ala-Tyr(Br)-Ala. (B) PepT$_{So2}$ in complex with Ala-Tyr(Br). Residue labels followed by an asterisk refer to residues in the peptide. The N- and C-termini of the peptide are indicated with boxes. (C) PepT$_{So2}$ in complex with Ala-Tyr(Br)-Ala. Same orientation as in panel B. (D) Surface representation of an overlay of PepT$_{St}$ in complex with Ala-Phe (PDB ID 4D2C) (light cyan) and Ala-Ala-Ala (PDB ID 4D2D) (dark cyan). The inset shows the different positions of the di- and tripeptides in the binding cavity, where Ala-Phe is oriented horizontally and Ala-Ala-Ala is positioned vertically. (E) PepT$_{St}$ in complex with Ala-Phe (PDB ID 4D2C). (F) PepT$_{St}$ in complex with Ala-Ala-Ala (PDB ID 4D2D). Same orientation

as in panel E. (G) Comparison of the position of the dipeptide backbone for the structure of $PepT_{St}$ in complex with Ala-Leu ($PepT_{St}$[Ala-Leu]) (PDB ID 5OXL), which is colored wheat, and $PepT_{St}$[Phe-Ala] (PDB ID 5OXN), which is light blue. The former represents dipeptide binding mode 1, while the latter represents binding mode 2. Binding site residues are shown in sticks, and the dipeptides are shown in cartoon representation. (H) Binding of the C-terminus of Ala-Leu in $PepT_{St}$[Ala-Leu]. Hydrogen bonds and salt bridges between the protein and the peptide C-terminus (distances 2.7 – 3.4 Å) are indicated by grey dashes. (I) Binding of the C-terminus of Phe-Ala in $PepT_{St}$[Phe-Ala]. The C-terminus is interacting indirectly through a water molecule (red sphere) with Arg-26 and Tyr-30 (distances 3.1 – 3.2 Å).

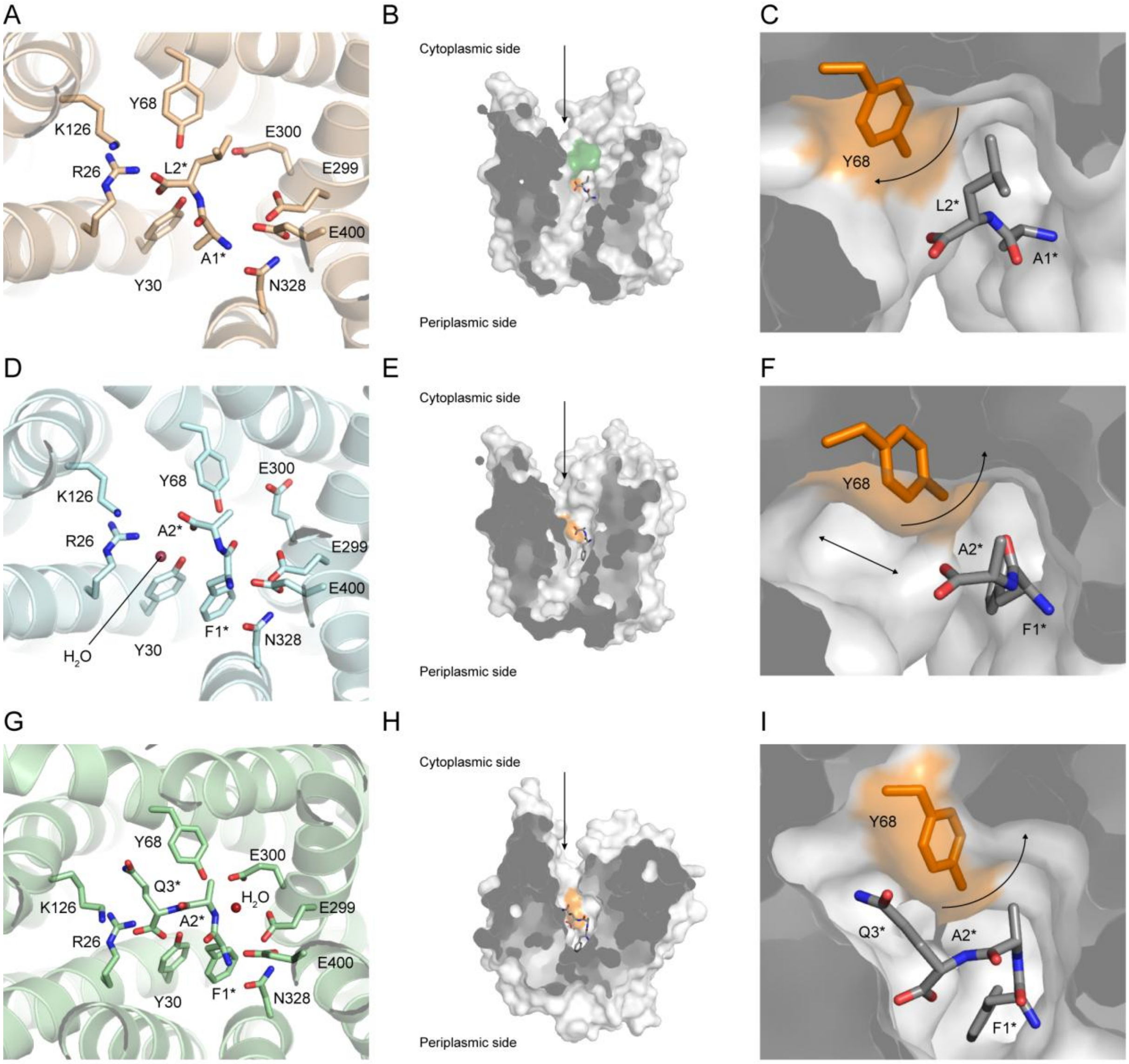

Figure S2: Role of Tyr-68 in di- and tripeptide binding in PepT$_{St}$. (A) Binding site of PepT$_{St}$[Ala-Leu] (PDB ID 5OXL), which represents dipeptide binding mode 1. (B) Surface representation of PepT$_{St}$[Ala-Leu]. The protein is colored white except for Tyr-68, which is orange, and for Phe-428 and Trp-427, which are green. The latter two residues partially restrict access to the binding site from the cytoplasmic side in this structure. The grey sticks represent the Ala-Leu dipeptide, and the arrow indicates the position from which the figure in panel C was generated. (C) Detailed position of Tyr-68 in the binding cavity when Ala-Leu is bound. The side chain of Tyr-68 is represented by sticks and labeled. The arrow indicates that the residue is in a position that widens one pocket (pocket 2), while restricting access to a neighboring one (pocket 3). (D) Binding site of PepT$_{St}$[Phe-Ala] (PDB ID 5OXN), representing dipeptide binding mode 2. (E) Surface representation of PepT$_{St}$[Phe-Ala]. (F)

Detailed position of Tyr-68 in the binding cavity when Phe-Ala is bound. The arrows indicate that Tyr-68 is in a position that narrows pocket 2, while allowing access to pocket 3. (G) Binding site of $PepT_{St}$[Phe-Ala-Gln]. (H) Surface representation of $PepT_{St}$[Phe-Ala-Gln]. (I) Detailed position of Tyr-68 in the binding cavity when Phe-Ala-Gln is bound. The position is similar to the one it adopts in $PepT_{St}$[Phe-Ala]. There is therefore access to pocket 3. Indeed, the C-terminal glutamine residue of the tripeptide is here occupying this pocket.

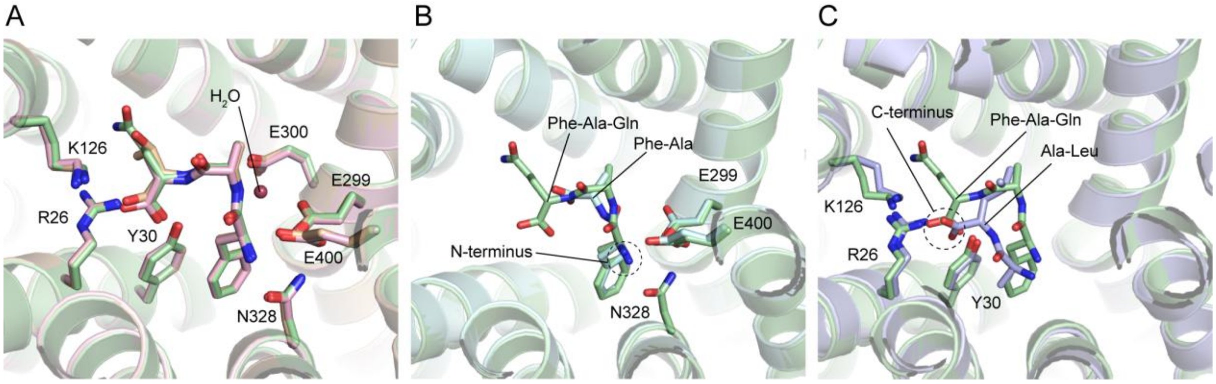

Figure S3: Binding mode of Phe-Ala-Gln compared to the binding modes of other peptides binding to PepT$_{St}$. (A) Comparison of the binding mode of Phe-Ala-Gln to the binding modes of Phe-Ala-Ala and Phe-Ala-Thr. PepT$_{St}$[Phe-Ala-Gln] is light green, PepT$_{St}$[Phe-Ala-Ala] is light pink, and PepT$_{St}$[Phe-Ala-Thr] is wheat. The first (Phe) and second (Ala) residues of the peptides show very strong overlap. To which extent the third residues overlap is however unclear, since the positions of these residues are ambiguous in PepT$_{St}$[Phe-Ala-Ala] and PepT$_{St}$[Phe-Ala-Thr] (see main text and Figure 2 A-D). (B) Comparison of the binding modes of Phe-Ala-Gln and Phe-Ala. PepT$_{St}$[Phe-Ala-Gln] is light green and PepT$_{St}$[Phe-Ala] is cyan. The peptides show a very strong overlap, and their N-termini are bound in the same manner. Residues coordinating the N-terminus of the peptide (in a circle) are represented in sticks. (C) Comparison of the binding modes of Phe-Ala-Gln and Ala-Leu. PepT$_{St}$[Phe-Ala-Gln] is light green and PepT$_{St}$[Ala-Leu] is light violet. The C-terminus of Phe-Ala-Gln is bound in a similar though not identical manner as observed for Ala-Leu. Residues coordinating the C-terminus of the peptide (in a circle) are represented in sticks.

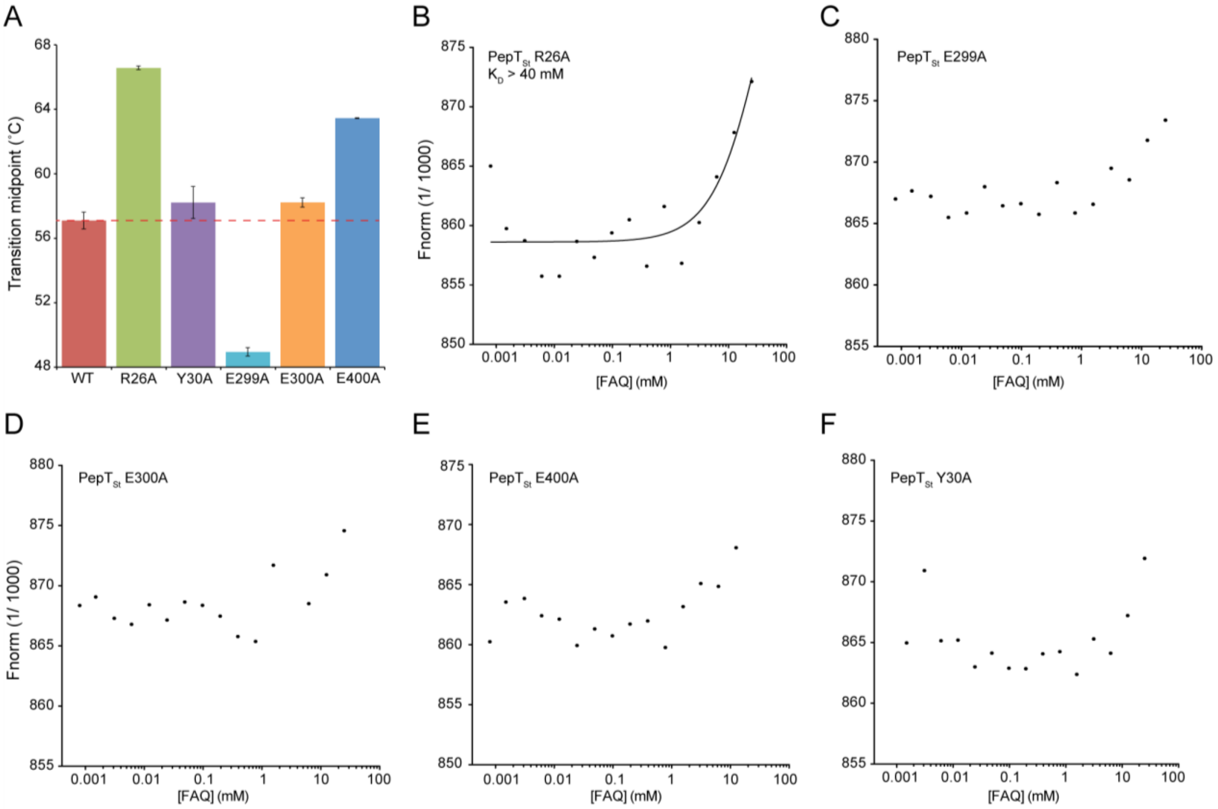

Figure S4: Stability and peptide binding of PepT$_{St}$ mutants. The following peptide binding site residues were mutated to alanine: Arg-26, Tyr-30, Glu-299, Glu-300 and Glu-400. (A) Stability measurements of the mutants in comparison to WT PepT$_{St}$. The Y30A and E300A mutants showed about the same thermal stability as WT PepT$_{St}$, whereas the R26A and E400A mutants were found to be significantly more stable, and the E299A mutant markedly less so. The dashed red line indicates the Tm for the WT protein. Each measurement was performed in triplicates. In the plot, the average value for the three independent measurements is shown together with the standard deviation associated with each mutant. (B–E) Microscale thermophoresis (MST) was used to characterize binding of Phe-Ala-Gln to the mutants. (B) Binding to PepT$_{St}$ R26A (the estimated K_D value is indicated). (C) Binding to PepT$_{St}$ E299A. (D) Binding to PepT$_{St}$ E300A. (E) Binding to PepT$_{St}$ E400A. (F) Binding to PepT$_{St}$ Y30A.
